# Usefulness of heart rhythm complexity in heart failure detection and diagnosis

**DOI:** 10.1038/s41598-020-71909-8

**Published:** 2020-09-10

**Authors:** Cheng-Hsuan Tsai, Hsi-Pin Ma, Yen-Tin Lin, Chi-Sheng Hung, Shan-Hsuan Huang, Bei-Lin Chuang, Chen Lin, Men-Tzung Lo, Chung-Kang Peng, Yen-Hung Lin

**Affiliations:** 1 Department of Internal Medicine, National Taiwan University Hospital JinShan Branch, New Taipei, Taiwan; 2 Department of Internal Medicine, National Taiwan University Hospital and National Taiwan University College of Medicine, 7 Chung-Shan South Road, Taipei, Taiwan; 3 Department of Electrical Engineering, National Tsing Hua University, Hsinchu, Taiwan; 4 Department of Internal Medicine, Taoyuan General Hospital, Taoyuan, Taiwan; 5 Department of Biomedical Sciences and Engineering, National Central University, Chungli, Taiwan; 6 Division of Interdisciplinary Medicine and Biotechnology, Beth Israel Deaconess Medical Center/Harvard Medical School, Boston, MA USA

**Keywords:** Applied mathematics, Scientific data, Diagnostic markers, Heart failure

## Abstract

Heart failure (HF) is a major cardiovascular disease worldwide, and the early detection and diagnosis remain challenges. Recently, heart rhythm complexity analysis, derived from non-linear heart rate variability (HRV) analysis, has been proposed as a non-invasive method to detect diseases and predict outcomes. In this study, we aimed to investigate the diagnostic value of heart rhythm complexity in HF patients. We prospectively analyzed 55 patients with symptomatic HF with impaired left ventricular ejection fraction and 97 participants without HF symptoms and normal LVEF as controls. Traditional linear HRV parameters and heart rhythm complexity including detrended fluctuation analysis (DFA) and multiscale entropy (MSE) were analyzed. The traditional linear HRV, MSE parameters and DFAα1 were significantly lower in HF patients compared with controls. In regression analysis, DFAα1 and MSE scale 5 remained significant predictors after adjusting for multiple clinical variables. Among all HRV parameters, MSE scale 5 had the greatest power to differentiate the HF patients from the controls in receiver operating characteristic curve analysis (area under the curve: 0.844). In conclusion, heart rhythm complexity appears to be a promising tool for the detection and diagnosis of HF.

## Introduction

Heart failure (HF) remains an important cause of morbidity and mortality and it also leads to a tremendous social burden worldwide^1,2^. Although there have been dramatic achievements and improvements both in medical and device treatments for HF in recent decades, the incidence of HF is still increasing^3,4^. The early diagnosis of HF still remains a challenge in the modern era. An easily performed, low cost and non-invasive tool would therefore be especially useful for the early detection and diagnosis of HF.

HF is a complex syndrome which involves neurohormonal activation and autonomic nervous system dysfunction, leading to overactivation of the sympathetic system and a reduction in vagal activity^5^. Heart rate variability (HRV) is a fundamental and non-invasive techniques used to assess autonomic modulation of the heart^6–9^. Traditional HRV has been shown to be markedly reduced in patients with HF^9,10^, and this reduction has been related to the severity of HF and its prognosis^11^. However, the predictive power of traditional linear HRV is unsatisfactory^12^. Accordingly, non-linear methods to analyze the complexity rather than only changes in the variability beneath the R-R intervals have been developed^13,14^. Heart rhythm complexity derived from non-linear HRV measures the complexity of heart rate interval, and is composed of multiscale entropy (MSE) and detrended fluctuation analysis (DFA). Compared to linear parameters, heart rhythm complexity has shown a much greater ability to detect diseases such as end-stage renal disease, primary aldosteronism, and pulmonary hypertension^15–17^. Therefore, it would seem to be reasonable to use heart rate complexity in the detection and diagnosis of HF. However, only few studies have investigated the role of heart rhythm complexity in predicting the prognosis of HF^12,18^, data on heart rhythm complexity in the detection of HF are lacking. Therefore, in this study, we aimed to evaluate the ability of heart rhythm complexity in the detection and diagnosis of HF.

## Methods

### Patients

We prospectively enrolled 55 Taiwanese patients with symptomatic HF with impaired left ventricular ejection fraction (LVEF < 50%) at National Taiwan University Hospital from August 2008 to December 2015. We also enrolled 97 participants who were admitted to our hospital and received coronary angiogram examinations which revealed patent coronary arteries or non-significant coronary artery disease (CAD; stenosis ≤ 50%) as the control group. The patients in the control group did not have any HF symptoms and had a LVEF > 55% at enrollment. Patients with chronic atrial fibrillation, uncontrolled arrhythmia and cardiac pacemaker paced rhythm were excluded from this study.

The baseline demographics, past medical history, complete blood count, biochemistry studies, and medications were recorded at enrollment. All subjects provided written informed consent for storage of their information in the hospital database and usage for research.

This study was approved by the Institutional Review Board of National Taiwan University Hospital and all research was performed under relevant guidelines and regulations.

### Echocardiography

All subjects received standard transthoracic echocardiography (iE33 xMATRIX Echocardiography System, Philips, Amsterdam, Netherlands). The LVEF was measured via a parasternal long axis view (M-mode) and apical 4-chamber view (biplane Simpson's method) in accordance with the recommendations of the American Society of Echocardiography^19^.

### Ambulatory ECG recording and assessment of HRV

All patients in this study received 24-h ambulatory ECG Holter (Zymed DigiTrak Plus 24-h Holter Monitor Recorder and DigiTrak XT Holter Recorder 24 h, Philips, Amsterdam, Netherlands) recordings. During the examination, all patients maintained their daily activities without specific limitations. Holter recordings in the HF patients were not performed during acute decompensated HF, and were only performed in patients with a stable condition. In the control group, Holter recordings were performed within 1 week after coronary angiography.

A stable 4-h segment of daytime RR intervals (between 9AM and 5PM) was selected. The selected RR intervals were automatically annotated using an algorithm, and then examined by two experienced technicians. MATLAB software with self-written code was used to process the signals. Traditional linear HRV analysis including time and frequency domain analysis, and non-linear HRV analysis including DFA and MSE were performed by independent physicists.

Traditional linear HRV analysis including time and frequency domain analysis was performed according to the recommendations of the North American Society of Pacing Electrophysiology and the European Society of Cardiology^20^. Time domain HRV parameters including mean RR, standard deviation of normal RR (SDRR), percentage of the absolute change in consecutive normal RR intervals exceeding 20 ms (pNN_20_) and percentage of the absolute change in consecutive normal RR intervals exceeding 50 ms (pNN_50_) were calculated. For the frequency-domain, spectral analysis was performed using fast Fourier transform algorithms to analyze high frequency (HF; 0.15–0.4 Hz), low frequency (LF; 0.04–0.15 Hz), and very low frequency (VLF; 0.003–0.04 Hz) in this study.

Non-linear HRV analysis, including MSE and DFA based on chaos and fractal theories, respectively, was used in this study as the heart rhythm complexity parameters. DFA provides an algorithm to reveal the fractal behavior beneath seemingly nonstationary RR dynamics and remove trends from integrated time series. DFA can also quantify the degree of self-affinity^14^. DFA was performed by summing the detrended integrated time series in each scale. Short DFAα1 (4–11 beats) and long DFAα2 (11–64 beats) values were obtained from the slope of the log–log plots of fluctuations against time scales.

MSE is used to calculate entropies or the complexities of physiological signals on multiple time scales^21^. MSE uses a coarse-graining process (i.e. averaging consecutive beats to form a new time series) to construct many different time scales, and it can be used to quantify the complexity of biological signals in different time scales^22^. In this study, the entropy values of scale 5 (scale 5), the linear-fitted slope of scale 1–5 (slope 5), the summation of entropy values of scales 1–5 (area 1–5) and 6–20 (area 6–20) were calculated to represent the complexity of the RR dynamics in short- and long-time scales.

### Statistical analysis

Data were expressed as mean ± standard deviation and median (25th and 75th percentiles) for normally distributed and non-normally distributed data, respectively. Comparisons of data between the patients with and without HF were made using the independent t-test and Mann–Whitney U test. Differences between proportions were assessed using the chi-square test or Fisher’s exact test. Logistic regression analysis was used to validate associations between variables and the presence of HF. Significant determinants in univariable logistic regression analysis (P < 0.05) including mean RR, SDRR, pNN_20_, VLF, LF, HF, LF/HF ratio, DFAα1, DFAα2, slope 5, scale 5, area 1–5 and area 6–20 were then tested in multivariable logistic regression analysis with a stepwise subset selection to identify independent factors to predict the presence of HF. In multivariable regression analysis, the independent HRV predictors of HF including pNN_20_, DFAα1, DFAα2 and MSE scale 5 were adjusted for clinical variables including age, sex, creatinine, fasting glucose, CAD, hypertension, diabetes mellitus (DM), dyslipidemia, and the use of beta blockers, calcium channel blockers (CCBs) and angiotensin II receptor blockers (ARBs) or angiotensin-converting enzyme inhibitors (ACEIs) in five different logistic regression models. The area under the curve (AUC) was used to assess the discriminatory power of the model to predict HF. All statistical analyses were performed using R software 3.5.1 (https://www.r-project.org/) and SPSS version 25 for Windows (SPSS Inc., IL, USA). The significance level of the statistical analysis was set at 0.05.

## Results

### Patients

The baseline clinical and echocardiographic data are shown in Table 1. The patients with HF had significantly higher prevalence rates of CAD, DM, ARBs or ACEIs usage, beta blocker usage, and a lower rate of CCBs usage. In addition, the HF patients had significantly higher serum creatinine and fasting glucose levels. The other variables were comparable between the two groups except for the echocardiographic data. The LVEF was 35 ± 9.4% in the HF patients and 70 ± 5.6% in the controls. The diameters of left ventricular end-diastolic diameter and left ventricular end-systolic diameter in the HF patients were also significantly higher compared with the controls.

**Table 1:** Clinical data of the patients.

	HF (N = 55)	Control (N = 97)	*P* Value
Age (years)	61 ± 14	59 ± 11	0.215
Male, n (%)	43 (78%)	65 (67%)	0.144
CAD, n (%)	41 (74)	9 (9%)	< 0.001
DM, n (%)	23 (42%)	23 (24%)	0.020
HTN, n (%)	31 (56%)	63 (65%)	0.295
Dyslipidemia, n (%)	15 (27%)	18 (19%)	0.08
**Medication**			
ACEI/ARB	44 (80%)	32 (33%)	< 0.001
Beta-blocker	39 (71%)	46 (47%)	0.005
CCB	9 (16%)	31 (32%)	0.036
Glucose AC, mg/dL	118 ± 38	100 ± 17	0.002
Creatinine, mg/dL	1.6 ± 1.5	0.93 ± 0.20	0.002
ALT	35 ± 43	24 ± 10	0.125
TG, mg/dL	151 ± 99	131 ± 68	0.192
T-Chol, mg/dL	181 ± 38	173 ± 36	0.239
LVEF, %	35 ± 9.4	70 ± 5.6	< 0.001
LVEDD, mm	60 ± 9.6	47 ± 3.8	< 0.001
LVESD, mm	50 ± 9.7	28 ± 3.0	< 0.001

Data were presented as mean ± standard deviation or number (percentage).

*CAD* coronary artery disease, *DM* diabetes mellitus, *HTN* hypertension, *ACEI* Angiotensin-converting enzyme inhibitors, *ARB* angiotensin receptor blockers, *CCB* calcium channel blocker, *TG* triglyceride, *T-Chol* total cholesterol, *LVEF* left ventricular ejection fraction, *LVEDD* left ventricular end-diastolic diameter, *LVESD* left ventricular end-systolic diameter.

### Linear and non-linear HRV analysis

**Figure 1:**
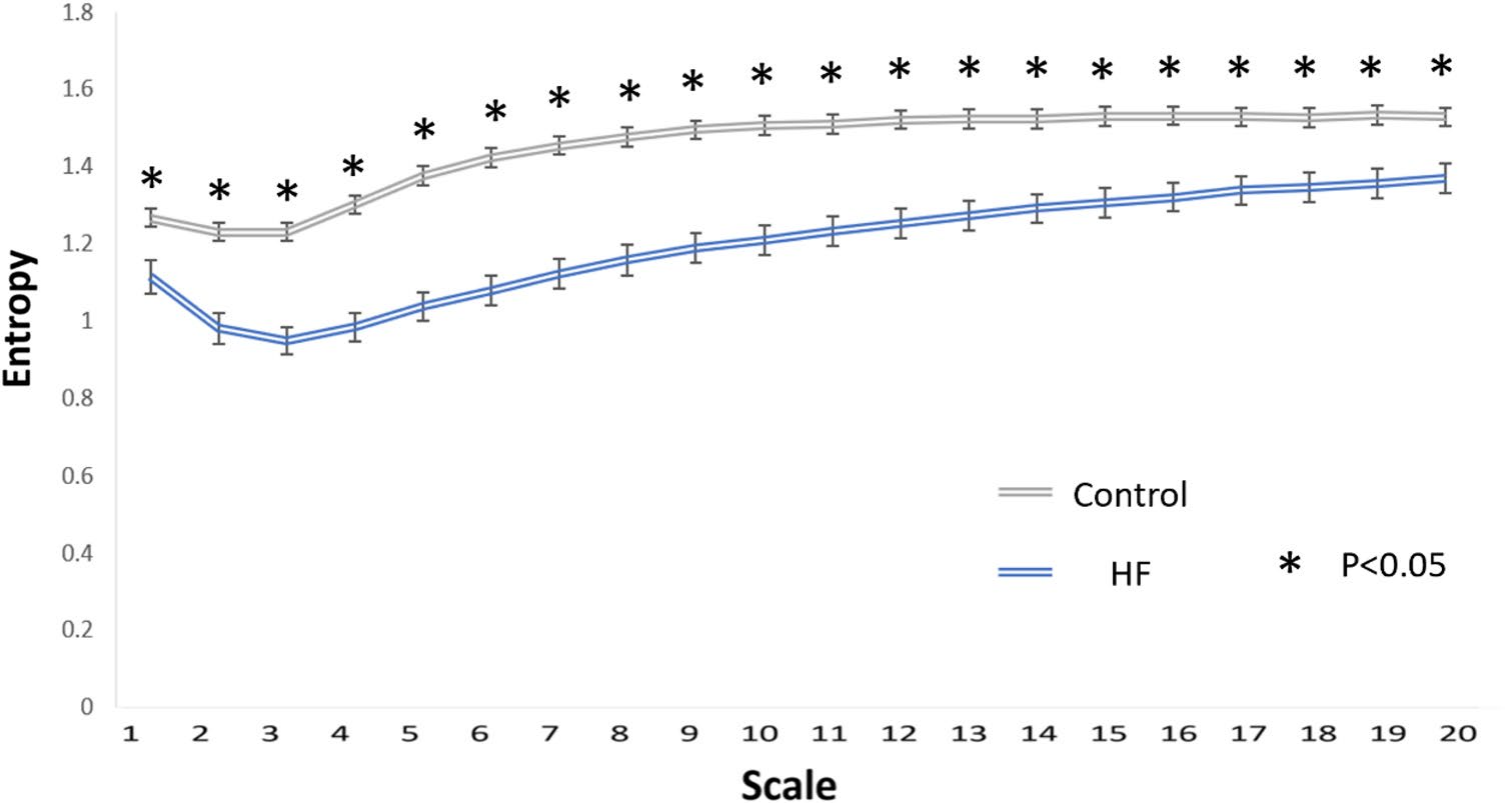
The entropy over different time scales in patients with (blue) and without (grey) heart failure. *p < 0.001.

All HRV parameters including linear HRV, MSE and DFA parameters were significantly lower in the HF patients compared with the controls, except for DFAα2 which was higher in the HF group (Table 2). The mean RR, SDRR, pNN_20_, pNN_50_, VLF, LF, HF, LF/HF ratio, DFAα1, MSE slope 5, scale 5, area 1–5 and area 6–20 were significantly lower in the patients with HF. In addition, the entropies of different time scales of MSE curves were significantly different between the HF patients and controls (Fig. 1).

**Table 2:** Holter Parameter in HF patients and control.

	HF (N = 55)	Control (N = 97)	*P* value
**Time domain analysis**			
Mean RR	740.59 (673.54 ~ 808.53)	841.55 (737.90 ~ 907.57)	< 0.001
SDRR	29.08 (22.01 ~ 27.22)	40.94 (33.11 ~ 48.21)	< 0.001
pNN_20_	0.17 (0.049 ~ 0.32)	0.32 (0.20 ~ 0.42)	< 0.001
pNN_50_	0.012 (0.0035 ~ 0.052)	0.029 (0.010 ~ .066)	0.023
**Frequency domain analysis**			
VLF	222.91 (128.43 ~ 382.66)	477.44 (308.03 ~ 644.04)	< 0.001
LF	47.51 (7.79 ~ 123.42)	157.34 (103.22 ~ 239.55)	< 0.001
HF	22.57 (10.39 ~ 53.15)	35.84 (24.58 ~ 80.05)	0.001
LF/HF ratio	2.77 (1.52 ~ 4.84)	4.64 (2.77 ~ 5.88)	< 0.001
**Detrended fluctuation analysis**			
DFAα1	1.03 (0.78 ~ 1.23)	1.25 (1.06 ~ 1.33)	< 0.001
DFAα2	1.23 (0.78 ~ 1.23)	1.16 (1.06 ~ 1.21)	0.005
**Multiscale entropy**			
Slope 1–5	− 0.0017 (− 0.048 ~ 0.024)	0.038 (− 0.013 ~ 0.077)	< 0.001
Scale 5	1.064 (0.86 ~ 1.14)	1.39 (1.24 ~ 1.52)	< 0.001
Area 1–5	3.88 (3.16 ~ 4.81)	5.23 (4.47 ~ 5.62)	< 0.001
Area 6–20	17.95 (15.44 ~ 20.31)	21.33 (19.33 ~ 22.92)	< 0.001

Data were presented as Values are median (25th–75th percentile).

*SDRR* standard deviation of normal RR intervals, *pNN*_*20*_ percentage of the absolute change in consecutive normal RR interval exceeds 20 ms, *pNN*_*50*_ percentage of the absolute change in consecutive normal RR interval exceeds 50 ms, *VLF* very low frequency, *LF* low frequency, *HF* high frequency, *DFA* detrended fluctuation analysis.

### Logistic regression analysis for predictors of HF

In univariable logistic regression, linear HRV parameters including mean RR, SDRR, pNN_20_, VLF, LF, HF and LF/HF ratio and all heart rhythm complexity parameters including DFAα1, DFAα2, MSE slope 5, scale 5, area 1–5 and area 6–20 were significantly associated with HF. These parameters were further analyzed using multivariable logistic regression, which showed that pNN_20_, DFAα1, DFAα2 and MSE scale 5 remained in the model as independent predictors of HF (Table 3). These four HRV parameters were adjusted for age, sex, creatinine, fasting glucose, CAD, hypertension, DM, dyslipidemia, and the use of beta blockers, CCBs and ARBs or ACEIs in different models. In model 5, only heart rhythm complexity parameters DFAα1 and MSE scale 5 remained as significant predictors of HF after adjustments (Table 4).

**Table 3:** Univariable and multivariable logistic regression model to predict the presence of heart failure.

	Univariable logistic regression		Multivariable logistic regression	
	β (95% CI)	P	OR (95% CI)	P
Mean RR	0.993 (0.989 ~ 0.996)	< 0.001		
SDRR	0.928 (0.898 ~ 0.959)	< 0.001		
pNN_20_	0.009 (0.001 ~ 0.096)	< 0.001	0.005 (< 0.001 ~ 0.202)	0.005
pNN_50_	0.065 (0.001 ~ 6.777)	0.249		
VLF	0.997 (0.996 ~ 0.999)	< 0.001		
LF	0.991 (0.987 ~ 0.995)	< 0.001		
HF	0.992 (0.984 ~ 1.000)	0.037		
LF/HF ratio	0.724 (0.607 ~ 0.863)	< 0.001		
DFAα1	0.039 (0.009 ~ 0.171)	< 0.001	0.021 (0.002 ~ 0.202)	0.001
DFAα2	760.450 (11.826 ~ 52,836.633)	0.002	< 0.001 (< 0.001 ~ 0.481)	0.030
Slope 5	< 0.001 (< 0.001 ~ 0.004)	< 0.001		
Scale 5	0.002 (< 0.001 ~ 0.015)	< 0.001	0.002 (< 0.001 ~ 0.038)	< 0.001
Area 1–5	0.283 (0.180 ~ 0.444)	< 0.001		
Area 6–20	0.697 (0.608 ~ 0.800)	< 0.001		

*In multivariable logistic regression, the mean RR, SDRR, VLF, LF, HF, LF/HF ratio, slope 5, area 1–5 and area 6–20 were excluded from the model.

*SDRR* standard deviation of normal RR intervals, *pNN*_*20*_ percentage of the absolute change in consecutive normal RR interval exceeds 20 ms, *pNN*_*50*_ percentage of the absolute change in consecutive normal RR interval exceeds 50 ms, *VLF* very low frequency, *LF* low frequency, *HF* high frequency, *DFA* detrended fluctuation analysis.

**Table 4:** Heart rhythm complexity to predict heart failure after adjustment.

	pNN_20_*	DFAα1*	DFAα2*	Scale 5*
Model 1	0.009 (0.001 ~ 0.096)^†^	0.039 (0.009 ~ 0.171)^†^	760 (11.83 ~ 5.3* 10^4^)^†^	0.002 (< 0.001 ~ 0.015)^†^
Model 2	0.008 (0.001 ~ 0.085)^†^	0.035 (0.007 ~ 0.175)^†^	1535 (18.64 ~ 1.3*10^5^)^†^	0.002 (< 0.001 ~ 0.016)^†^
Model 3	0.001 (< 0.001 ~ 0.018)^†^	0.025 (0.004 ~ 0.160)^†^	7,691 (49.05 ~ 1.3*10^6^)^†^	0.001 (< 0.001 ~ 0.028)^†^
Model 4	0.015 (0.001 ~ 0.257)^†^	0.093 (0.014 ~ 0.621)^†^	632 (2.74 ~ 1.5*10^5^)^†^	0.004 (< 0.001 ~ 0.040)^†^
Model 5	0.042 (0.001 ~ 1.977)	0.010 (< 0.001 ~ 0.199)^†^	1846 (0.80 ~ 4.2*10^6^)	0.005 (< 0.001 ~ 0.104)^†^

Data were presented with β (95% CI).

^†^P < 0.05 *Independent predictors of heart failure in multivariable logistic regression model including mean RR, SDRR, VLF, LF, HF, LF/HF ratio, DFAα1, DFAα2, slope 5, scale 5, area 1–5 and area 6–20 after stepwise subset selection.

Model 1 unadjusted.

Model 2 adjusted by age and sex.

Model 3 adjusted by age, sex, beta blocker, CCB and ARB or ACEI use.

Model 4 adjusted by age, sex, creatinine and AC glucose.

Model 5 adjusted by age, sex, creatinine, AC glucose, CAD, DM, HTN and dyslipidemia.

*pNN*_*20*_ percentage of the absolute change in consecutive normal RR interval exceeds 20 ms, *DFA* detrended fluctuation analysis.

### Comparisons of all linear HRV and heart rhythm complexity parameters to differentiate the patients with HF and controls

Among all HRV parameters, the MSE scale 5 had the highest AUC to predict the presence of HF among all HRV parameters in receiver operating characteristic (ROC) curve analysis (AUC: 0.844).

**Figure 2:**
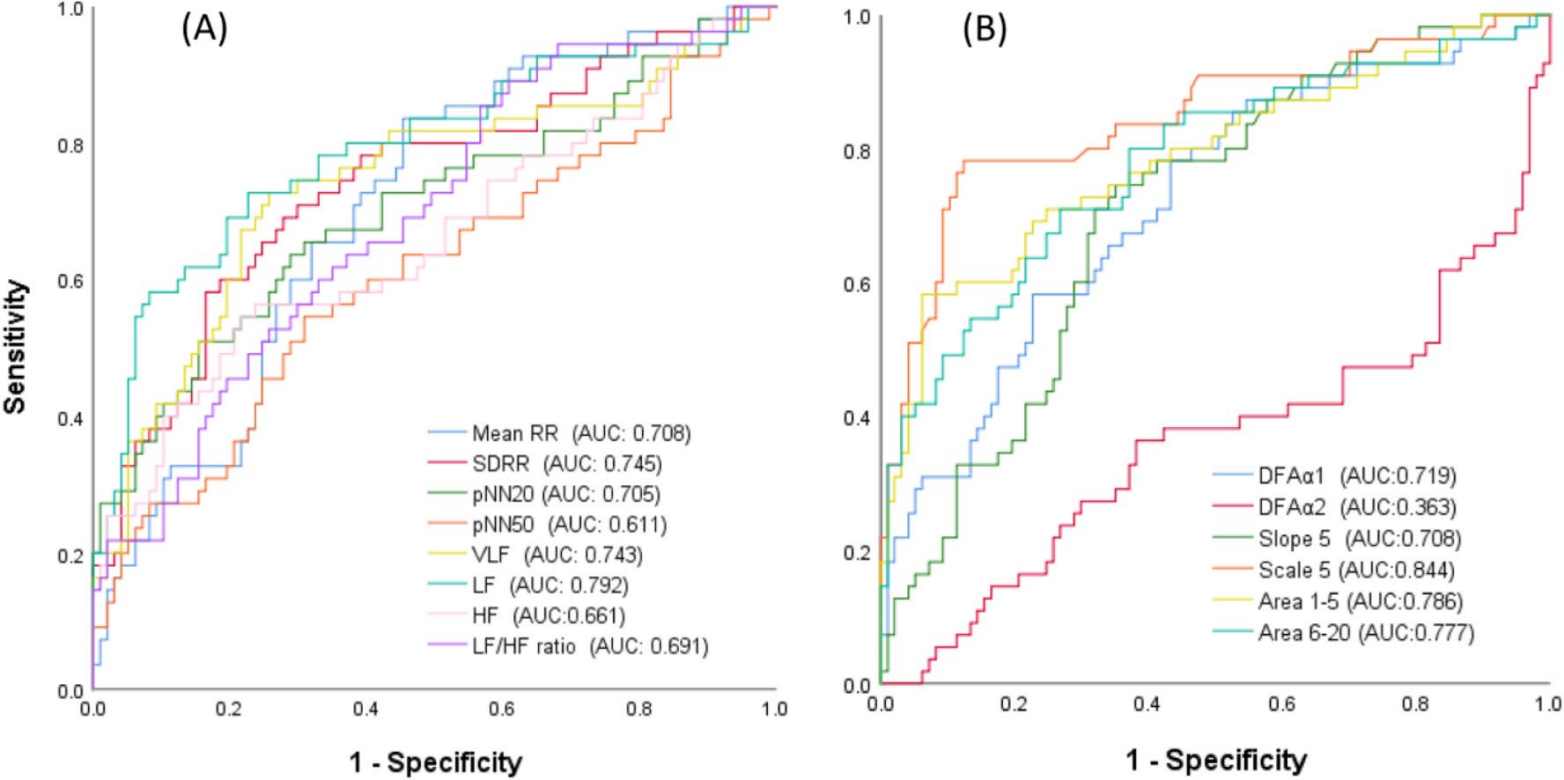
Analysis of the discriminatory power of the two groups in receiver operating characteristic curve analysis. (**A**) Shows the AUC of the linear heart rate variability parameters, and (**B**) shows the non-linear heart rate variability parameters that could discriminate the heart failure and control groups.

The AUCs of linear and HRV parameters including mean RR, SDRR, pNN_20_, pNN_50_, VLF, LF, HF and LF/HF ratio were 0.708, 0.745, 0.705, 0.611, 0.743, 0.792, 0.661 and 0.691, respectively, (Fig. 2A). The AUCs of non-linear and HRV parameters including DFAα1, DFAα2, slope 5, scale 5, area 1–5 and area 6–20 were 0.719, 0.363, 0.708, 0.844, 0.786 and 0.777, respectively, (Fig. 2B).

## Discussion

There are three major findings in this study. First, the patients with HF had both worse linear and non-linear HRV parameters compared to the healthy controls. Second, DFAα1 and MSE scale 5 were independent predictors of HF after adjusting for clinical variables. Third, MSE scale 5 had the greatest single discriminatory power to detect the presence of HF among all HRV parameters.

HF is one of the main causes of cardiovascular mortality and hospitalization in adults^4^. The prevalence of HF is estimated to be about 6.2 million in America and more than 23 million people worldwide^3,23^. In addition, the prevalence of HF has been predicted to increase by 46% from 2012 to 2030, so that more than 8 million Americans are expected to have HF by 2030^3^. HF is a complex syndrome resulting from impaired systolic and/or diastolic function with symptoms of dyspnea, fatigue and fluid retension^24^. Over the past decades, there has been tremendous progress in the treatment of chronic HF, however, chronic HF is still associated with an annual mortality rate of up to 15.6%^25^. Making an early diagnosis is extremely important to allow for timely interventions in these patients. Unfortunately, many patients do not seek medical assistance even after the development of symptoms in the early stage of the disease due to the non-specific or subtle presentations^26^. Since that, developing a useful diagnosis tool is urgently needed to improved outcomes. HRV has the potential to improve the diagnosis of HF and patient management.

The traditional linear HRV has been extensively studied in HF and autonomic dysfunction. In 1988, Saul et al. reported that severe HF patients had worse time domain and frequency domain HRV compared with healthy controls^9^. Binkley et al. demonstrated that parasympathetic withdrawal was a feature of HF^27^. Further studies also revealed that linear HRV were associated with volume status, functional class of HF, LVEF, serum norepinephrine and tumor necrosis factor level in HF patients^28–31^. Furthermore, functional test and the alternation of heart loading condition also interfere the diagnostic power of HRV. AR Kiselev et al. showed that frequency domain, LF analysis was superior to time domain analysis, SDRR during loading condition in HF detection^32^. The early appearance of these HRV abnormalities suggests that autonomic dysfunction plays a critical role in the progression of HF and HRV can be used in multiple serial tests during follow-up, and also that it has advantages in continuous disease monitoring. Patel et al. also showed that abnormal HRV was associated with the development of new HF^33^. However, previous HRV studies have mainly focused on linear analysis, and its ability to predict outcomes remains limited compared with non-linear HRV^8,12^. Non-linear HRV has also been studied with regards to the diagnosis and outcome prediction in many diseases with excellent results, including the outcomes of acute stroke^34^, primary aldosteronism^16^, severity of abdominal aorta calcification^35^, critical illnesses requiring extracorporeal life support^36^, post-myocardial infarction heart function^37^, and pulmonary hypertension^17^.

Heart rhythm complexity based on non-linear methods, including DFA and MSE measures the complexity beneath seemingly stationary biological signals^14,21^. A healthy biological system is able to make rapid adjustments to cope with dynamic environmental changes. This requires complex connections among autonomic, endocrine and cardiovascular systems. Once a subject is diseased, the system breaks down and the complexity decreases. Heart rhythm complexity analysis can quantify this phenomenon beneath biological signals instead of only variability provided by traditional linear HRV^13,14,21,38^. In the DIAMOND-CHF trial, the non-linear analysis parameter, DFAα1 but not linear parameters remained as an independent predictor of mortality in HF patients^12^. Patel et al. also recently demonstrated that abnormal DFAα1 in asymptomatic HF patients was associated with the onset of HF years in advance of the first clinical event in a retrospective analysis^33,39^. However, very few studies have investigated MSE analysis in HF patients. MSE was first introduced by Costa et al. in 2002^38^. In that paper, they demonstrated different patterns of MSE in patients with cardiovascular diseases such as atrial fibrillation and HF, however they retrieved ECG data from an ECG databank (physionet) without details of clinical variables. In addition, they did not analyze which MSE parameters can be used to detect HF, which limited its clinical application. Signorini et al. provided data supporting that HF patients had worse heart rate complexity; however, they only used the slope of the MSE curve as the MSE parameter. In our previous studies, we showed that individual scale values or areas under a certain serial scale could provide better power to detect a disease and predict the prognosis^15–17,35,36,40^. In addition, Signorini et al. did not compare non-linear methods to traditional linear methods^18^. In this study, we showed the advantage of heart rhythm complexity analysis over traditional linear methods, which may justify its use in clinical practice. In recent years, machine learning has been used to improve the diagnostic power of HRV by feature selection from time domain, frequency domain and non-linear analysis to form a new tool for HF detection^41–43^. However, the non-linear HRV feature which these studies used were mainly approximate entropy and sample entropy instead of multiscale entropy. Through coarse graining process, multiscale entropy can generate entropy in different time scales and provided more usefully information. In a recent small pilot study, our group showed that MSE could be used to predict survival in HF patients^40^, which further highlights the importance of MSE in cardiovascular diseases. In the future, we can integrate the MSE into the machine learning process to further improve the clinical use of HRV in HF detection.

In the current study, we found that the HF patients had significantly worse linear and non-linear HRV compared with the healthy controls, and that MSE scale 5 and DFAα1 were significant predictors of HF even after adjustments for multiple clinical variables. Furthermore, MSE scale 5 was the best single predictor of HF among all of the HRV parameters. These findings support our hypothesis that non-linear HRV has a better diagnostic power for HF compared with traditional linear HRV.

There are several limitations to this study. First, this is a small study with a limited number of cases which may have interfered with the statistical power of the analysis. The results of this study should be confirmed in larger clinical studies. Second, the data in the HF group were only derived from HF patients with LVEF < 50%, and patients with HF with preserved ejection fraction were not enrolled in this study. Third, the baseline characteristics including prevalence of CAD, DM and medications were different in the HF patients and controls, and they may still be confounders in this study. Fourth, functional tests were not included in this study which might improve the diagnostic power of HRV analysis. Fifth, this is a cross-sectional study. Further studies are needed to evaluate the prognostic value of heart rhythm complexity in HF patients.

## Conclusion

In conclusion, heart rhythm complexity had good diagnostic value in the enrolled patients with HF, and MSE scale 5 had the greatest single discriminatory power among all of the HRV parameters. The heart rhythm complexity had good potential for improvement in HF diagnosis and management.
